# Evaluation of a Vision-Guided Shared-Control Robotic Arm System with Power Wheelchair Users

**DOI:** 10.3390/s25154768

**Published:** 2025-08-02

**Authors:** Breelyn Kane Styler, Wei Deng, Cheng-Shiu Chung, Dan Ding

**Affiliations:** 1 Human Engineering Research Laboratories, VA Pittsburgh Healthcare System, Pittsburgh, PA 15206, USA; wed56@pitt.edu (W.D.); joshua.chung.cs@pitt.edu (C.-S.C.); dad5@pitt.edu (D.D.); 2 Department of Rehabilitation Science and Technology, University of Pittsburgh, Pittsburgh, PA 15206, USA

**Keywords:** assistive technology, robotic arm, mixed methods, usability

## Abstract

Wheelchair-mounted assistive robotic manipulators can provide reach and grasp functions for power wheelchair users. This in-lab study evaluated a vision-guided shared control (VGS) system with twelve users completing two multi-step kitchen tasks: a drinking task and a popcorn making task. Using a mixed methods approach participants compared VGS and manual joystick control, providing performance metrics, qualitative insights, and lessons learned. Data collection included demographic questionnaires, the System Usability Scale (SUS), NASA Task Load Index (NASA-TLX), and exit interviews. No significant SUS differences were found between control modes, but NASA-TLX scores revealed VGS control significantly reduced workload during the drinking task and the popcorn task. VGS control reduced operation time and improved task success but was not universally preferred. Six participants preferred VGS, five preferred manual, and one had no preference. In addition, participants expressed interest in robotic arms for daily tasks and described two main operation challenges: distinguishing wrist orientation from rotation modes and managing depth perception. They also shared perspectives on how a personal robotic arm could complement caregiver support in their home.

## 1. Introduction

Power wheelchair users with limited hand and arm function face challenges with reach [1] and independent manipulation tasks [2]. One option for enhancing this support are wheelchair-mounted assistive robotic manipulators which have been shown to increase independence and improve quality of life [3,4,5,6,7].

A large amount of research has made algorithmic advances in intuitive assistive robotic manipulator (ARM) control, specifically focusing on improving manual joystick operation. Traditionally, robotic arm control requires switching between multiple control modes [3,8]. For example, one mode moves the robotic arm in an x-y plane, another for z-axis movement and rotating the wrist, and additional modes for wrist orientation and gripper control. Previous work has shown that a large amount of task execution time is spent sequentially selecting modes to complete even simple robotic arm tasks. Additionally, even basic automatic time-optimal mode switching [9] or probabilistic language-grounded selection approaches [8] can improve operational efficiency.

Further improvements on manual operation focus on challenges in mapping the low dimensional user input (joystick axes) to high dimensional actions of the robotic arm. One category of related work addresses this by learning the mapping through a latent action space that can be directly controlled by the joystick [10]. Another category blends shared autonomy, combining the user’s joystick input with software assistance to simplify action execution. This includes recent work that integrates user expertise and task performance to dynamically adjust the level of assistance [11]. These works often emphasize users’ desire to maintain control, which has led to expanded research on when and where to provide additional assistance. For example, some studies allow users to rank suggested control mappings during teleoperation to reflect their customization preferences [12]. More recent systems combine blending autonomy with a latent intent space, learning shared autonomy strategies that leverage task repetition to determine when control should shift between the user and the robot. Specifically, control shifts back to the user during unseen tasks [13]. Collectively, these works reflect a growing focus on incorporating user preferences and subjective feedback into the evaluation of shared assistive systems. While these previous methods offer promising directions, they are limited to a single case study or lack testing with the intended user population. Many of these previous studies also focus on simplified single-step tasks rather than long-horizon multi-step tasks [14,15,16].

This work builds on a previously developed vision-guided shared (VGS) control system [17]. While the prior study focused on system development, this current study emphasizes evaluation with power wheelchair users. Our approach uses a shared control framework, which differs from the blending techniques described earlier. Shared control combines user inputs with autonomous system behavior, but is driven by the user. This is distinct from shared autonomy where software autonomy decides when to drive the task with limited user input [18]. In our VGS approach, the user performs gross movements of the robotic arm using a joystick and decides when to initiate autonomous actions during task execution. This is achieved by moving a wrist-mounted camera toward fiducial tags placed in the environment, which correspond to object interactions. When the tag is aligned, a circle is superimposed over it in the camera view on a touchscreen. Then the user can press the touchscreen circle to initiate the autonomous action, preserving a sense of control. This user-driven model was motivated by prior work on perceptions of autonomy and control in assistive devices [14,19,20,21,22,23]. These studies suggest that performance gains alone are insufficient if they come at the expense of user comfort or perceived agency.

Our study investigates user preferences and performance using the VGS system compared to manual joystick control across two functional kitchen tasks: a drinking task with six subtasks and a popcorn-making task with ten subtasks. Participants alternated between VGS and manual modes, and both quantitative performance metrics and qualitative feedback were collected. Notably, our findings suggest that performance metrics alone do not predict preference. We explore which subtasks presented the greatest difficulty and share user insights.

## 2. Materials and Methods

This study was approved as minimal-risk under the United States Veteran Affairs IRBNet protocol number 1617363. The in-lab study protocol consists of questionnaires, a training phase on the robotic arm, a testing phase comparing two different controls (VGS with manual joystick control) for two separate tasks (a drinking task and a popcorn making task), and then an exit interview. Participants were also encouraged to think aloud while performing the tasks.

### 2.1. Participant Selection

All participants went through a phone screening for eligibility and verbal consent. Eligible participants were then scheduled for on-site visits and sent a demographic survey which included demographic information and their perspectives on technology.

The inclusion criteria were: (1) 18 years of age and older; (2) using a power wheelchair as primary means of mobility; (3) having self-reported difficulties in performing everyday manipulation tasks such as reaching for a glass of water, opening a refrigerator, and picking up a toothbrush. The exclusion criteria were: (1) people with impaired vision; and (2) people with pressure ulcers that prevent them from sitting continuously for an extended period of time.

### 2.2. Sample Size Estimation

Based on our preliminary work, the VGS control could reduce manual task completion time by an average of 30% [24]. The functional tasks (which include the six-step drinking task and ten-step popcorn task) contain more complex tasks than previous work which focuses on single-step actions, thus we can reasonably assume at least 20% reduction in task completion time. Given an alpha of 0.05, a 20% reduction in task completion time, and a 25% standard deviation in the difference between the two interventions, we will need 12 participants to obtain 80% power for the study. Therefore, we aimed to recruit 16 participants to account for attrition. Power analysis was performed using G*power 3.1.9.2.

### 2.3. Task Description

**Figure 1:**
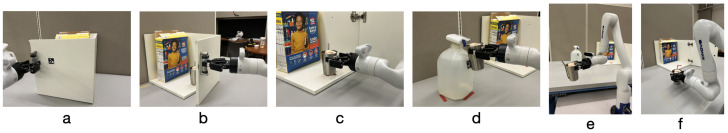
Steps of drinking task: (**a**) Grasp cabinet handle. (**b**) Open cabinet. (**c**) Grasp drink cup. (**d**) Fill drink cup and retract. (**e**) Bring cup to drink. (**f**) Place drink cup on table.

**Figure 2:**
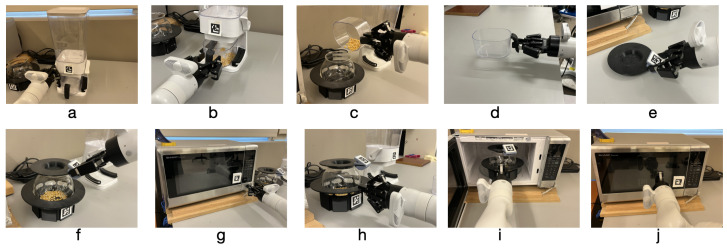
Steps of popcorn task: (**a**) Dispense popcorn kernels.(**b**) Grasp popcorn cup. (**c**) Pour kernels in jug.(**d**) Place popcorn cup on table. (**e**) Grasp jug lid. (**f**) Place lid on jug. (**g**) Open microwave. (**h**) Grasp jug. (**i**) Place jug in microwave. (**j**) Close microwave.

Users performed two separate tasks with the robotic arm. One was a drinking task consisting of six steps, Figure 1. It requires grasping the handle of the cabinet and opening the cabinet door. Then grasping a cup, bringing it to the water jug to fill, then bringing it back towards their face to simulate taking a drink. The task is completed when they then return the cup to the table. The second was a popcorn making task which consists of ten steps as shown in Figure 2. The robot arm is moved above a lever that is pushed down to dispense popcorn kernels into a small cup. Then the cup is grasped, popcorn kernels are poured into a microwave safe jug, and the small cup is returned to the table. A perforated lid is placed on the top of the jug to keep the popcorn from coming out. The task is complete after the user opens the microwave, places the jug in, and then closes the microwave. The lid and the jar holder are 3D printed to facilitate grasping.

### 2.4. Study Environment

**Figure 3:**
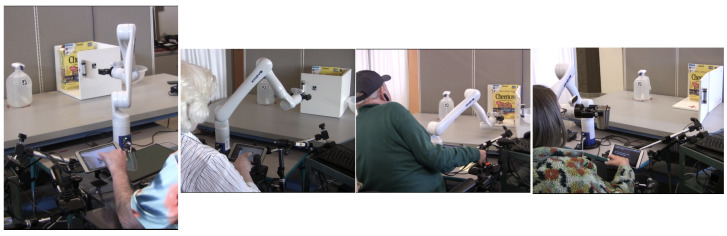
Power wheelchair users during the drinking task.

**Figure 4:**
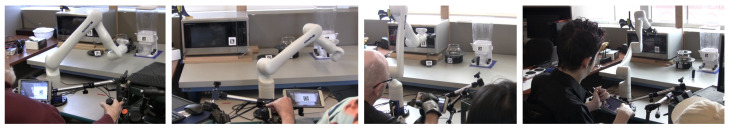
Power wheelchair users during the popcorn task.

During task execution, a Kinova Gen3 Robotic arm was clamped to a table and controlled through a two-axis joystick, Ablenet Buddy Button, and touchscreen. The joystick and touchscreen were mounted using quick-release lever attachments, allowing for easy adjustment and removal. Participants had two physical options for mode switching: one is to press the button on top of the robotic arm joystick and the other is to use an Ablenet Buddy Button. The button is necessary to switch between the four different arm modes (x-y, z and wrist rotation, wrist orientation, and open-close gripper). The setup was consistent for all participants. Participants positioned their wheelchairs to the left or right of the robotic arm based on their preference for joystick and touchscreen placement, which was adjusted according to their dominant hand used to control their power wheelchair. Pictures of power wheelchair users performing the task with the study setup are shown for the drinking task in Figure 3 and the popcorn making task in Figure 4.

The robotic arm includes a wrist mounted camera that is used to detect fiducial tags in the environment. These tags provide a six degree of freedom position in the world frame and an identification number which are tied to object properties as part of the system state. The position is used to generate trajectories for best grasp interaction with the objects. Additional details on the development of the system are provided in our previous study [17]. Since VGS is a shared control platform, the user and software take turns at various points in the task. In this shared control system the user operates the gross motions with the joystick and then initiates a switch to autonomous software control which executes the fine manipulation portions of the task. The architecture and baseline evaluation of the system are further described in previous work [17,25].

**Figure 5:**
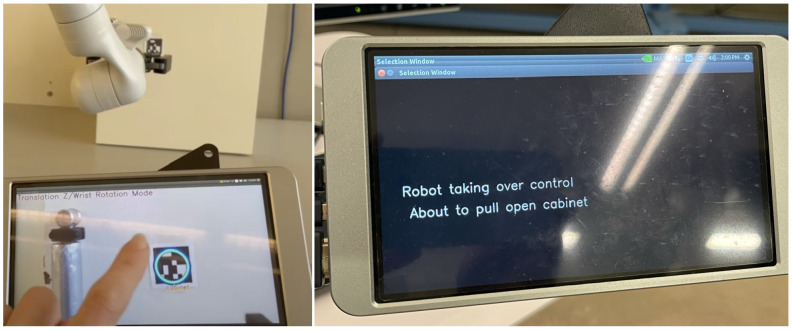
User selecting the touchscreen to switch to autonomous control for opening the cabinet. The screen then displays status information.

The touchscreen was only used during VGS control and showed a livestream of the robotic arm’s wrist-mounted camera. When users move the robotic arm towards an object for interaction, a circle appears on the touchscreen, shown in Figure 5, superimposed over the fiducial tag of that object. Tapping the circle initiates the autonomous portion of the task. The touchscreen then displays feedback on the task currently being executing, also shown in Figure 5. Once the task is complete, control is either returned to the user by showing the camera’s livestream, or the touchscreen waits for the user to finish their portion of the task and tap the screen to continue. Not all subtasks are completed autonomously by the system. The order of turn-taking and delegation of control within each subtask is shown in detail in Table 1.

### 2.5. Study Protocol

After providing informed consent, participants completed the Montreal Cognitive Assessment (MoCA). A cut-off score of 12 was used to determine adequate cognitive function for study participation [26]. Then, they completed a demographic questionnaire that included technology preferences, such as ranking factors like safety, cost, and aesthetics, as well as participants’ attitudes toward technology. They also completed the Spinal Cord Injury-Functional Index (SCI-FI) self-care short form to assess their ability to perform daily self-care activities, many of which involve upper limb manipulation [27].

#### 2.5.1. Training

Participants went through a two step training procedure. In the first training session, participants learned basic operation, including four control modes: X-Y mode for horizontal movement; Z direction and wrist rotation mode for vertical movement and wrist roll; wrist orientation mode for yaw and pitch wrist movement; and finger mode for opening and closing the gripper. Participants then practiced basic movements with the ARM (i.e., moving the ARM to table, positioning it on top of a box, picking up a cup, putting the cup back to the top of the box). At the end of the training, a pretest was performed to determine participants’ eligibility for the subsequent experimental test procedures. Participants were asked to perform three simple tasks on a task board including pressing a large round button, flipping a toggle switch, and pushing down a door handle [28]. If participants successfully completed each task within two minutes, they proceeded to the main study. Otherwise, additional training and practice was provided.

The second training session introduced users to the VGS control with fiducial markers. Users were able to practice subtasks and ask system clarification questions.

#### 2.5.2. Testing Protocol

Participants were asked to complete two tasks, i.e., a drinking task and a popcorn-making task, across two study visits, with one task per visit. Each visit lasted no more than three hours. For each task, participants first performed two trials using VGS control, followed by two trials using manual control. Therefore, the study consisted of two site visits resulting in a total of eight trials. During each trial, performance metrics were recorded. Then following each control mode, usability and perceived workload were recorded.

At the end of the second visit, participants took part in a brief semi-structured interview (∼20 min) led by study staff to discuss their experiences, perceptions of VGS versus manual control, and suggestions for improvement.

## 3. Data Collection and Analysis

We collected both quantitative and qualitative data. Quantitative measures included task performance metrics, standardized usability assessments, and responses to a demographic survey. Qualitative data was collected by capturing user perspectives during control operation and a semi-structured exit interview.

The demographic survey gathered participant information on injury level, wheelchair use, and prior experience with assistive and general technologies. As part of this survey, functional independence was assessed using the Spinal Cord Injury Functional Index Self-Care Short Form (SCI-FI SF), an 11-item validated measure of self-care ability in individuals with spinal cord injury [29]. With example questions such as, “Are you able to shampoo your hair?”, “Are you able to brush your teeth?”, and “Are you able to wash and dry your body?”, etc. Participant scores were converted to T-scores (mean = 50, SD = 10) for standardization and comparison.

The survey also included a set of items addressing attitudes toward technology, including participants’ comfort with and exposure to assistive and mainstream technologies. These responses were visualized using violin plots to illustrate distributions across the sample.

Following each control mode (VGS and manual), participants completed the System Usability Scale (SUS) and the NASA Task Load Index (NASA-TLX) to evaluate usability and perceived workload of the control mode. In total, each participant completed four SUS and four NASA-TLX assessments.

The SUS consists of ten items rated on a 5-point Likert scale from strongly disagree to strongly agree [30]. The items alternate between positive and negative phrasing to reduce response bias. Scores for positively worded items were computed as the scale position minus one, while scores for negatively worded items were calculated as five minus the scale position. The final SUS score was obtained by summing the adjusted item scores and multiplying by 2.5, resulting in a score out of 100. Higher scores reflect greater usability and overall satisfaction with the system.

The NASA-TLX was administered to assess subjective workload across six dimensions: mental demand, physical demand, temporal demand, performance, effort, and frustration. Each dimension was scored on a visual analog scale ranging from 0 to 100 in increments of five. The final workload score for each participant was calculated as the average of the six sub-scale scores. The NASA-TLX is a well-validated instrument [31].

All quantitative analyses were conducted using IBM SPSS Statistics (Version 29.0.2.0). Comparisons between conditions for the SUS and NASA-TLX scores were made using the Wilcoxon Signed-Rank Test, a non-parametric test appropriate for within-subject comparisons. A significance level of 0.05 was used for all statistical tests.

Performance metrics were also completed after each trial. Task performance was evaluated based on task completion times and success rates. These metrics were summarized using descriptive statistics to assess variation and trends across study conditions. A maximum duration of five minutes was assigned to each subtask, shown in Table 1, based on prior bench-top testing. If a subtask exceeded this time limit, participants were asked to stop and move on. Success was defined as completing the subtask within the five-minute time frame. Participants were allowed to attempt the task as many times as possible until they succeeded or the maximum time was reached.

Qualitative feedback was noted during task execution and collected through interviews. We used a deductive coding approach, with codes derived from the interview guide which asked about perceived ARM usage and their preferred control method. Responses were transcribed and analyzed in NVivo. Codes included: ARM usage for daily activities, ARM versus caregiver assistance, and control mode preferences. Results are grouped by code with representative quotations selected to illustrate key participant perspectives. Because participant responses were often brief and structured as direct quotations, we did not perform a full thematic analysis. Instead, illustrative quotations were selected to highlight key user insights and support the interpretation of quantitative findings. These quotes are presented in the results as narrative takeaways to contextualize outcomes.

## 4. Results

### 4.1. Demographics

All participants in the study were novice users. A total of 21 individuals were recruited, but only 12 completed the revised protocol. The first five participants were used to refine the study protocol. As procedural adjustments were made during this early phase, their data were not collected under consistent conditions and were therefore excluded from the final analysis. Four additional participants dropped out due to last minute scheduling conflicts or personal health issues. Demographic information for the 12 participants who completed the study is presented in Table 2.

The second column presents the user’s diagnosis, and the third column their age. The last column shows the user’s functional SCI-FI score which characterizes their upper limb impairment. A score greater or equal to 61.2 indicates no problem, a score from 51.3 to 61.2 indicates mild function deficit, a score of 51.3 to 41.2 indicates moderate function deficit, and a score less than 41.2 indicates severe function deficit. Based on the SCI-FI T-score, there was a spread of functional abilities where two indicated no problem, three had mild deficit, four had moderate deficit, and three exhibited severe deficit.

#### Attitude Towards Technology

**Figure 6:**
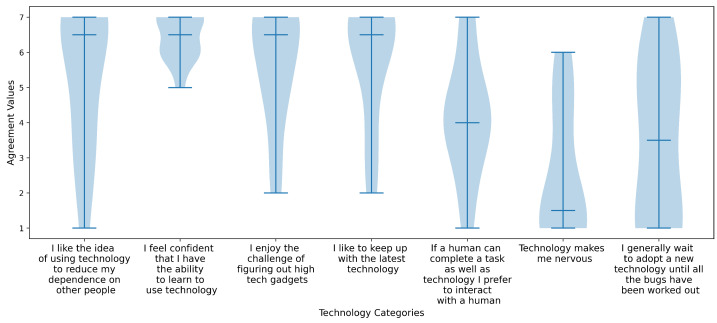
This figure shows the users attitudes towards technology categories. The middle line in the violin plot indicates the median value and the width of each plot represents the number of users that answered that agreement level. For example, all users had an agreement level of 5 or higher for, “I feel confident that I have the ability to learn to use technology.”

The following figures illustrate participants’ perceptions of technology, represented as violin plots. These plots display greater width in areas with a higher density of responses and narrower width where fewer responses were recorded. Participants’ attitudes toward technology are shown in Figure 6. They were asked to respond using a 7-point Likert scale, where 1 indicated strong disagreement and 7 indicated strong agreement with various technology-related statements shown on the x-axis. Overall users liked the idea of using technology to reduce independence, including enjoying the challenge of figuring things out and keeping up with latest trends. All felt confident they have the ability to learn to use technology. However, responses were widespread on if users would prefer human assistance over technology, and if they would be willing to adopt technology after all of the bugs are worked out.

**Figure 7:**
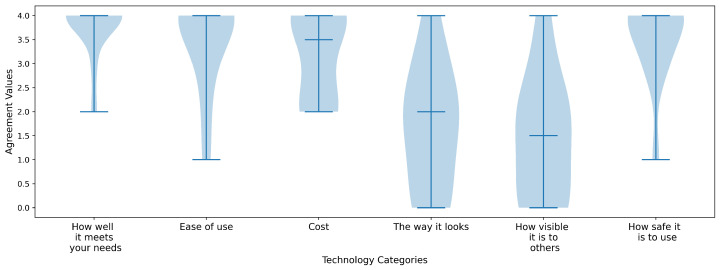
This is a violin plot showing users agreement levels on how important technology categories are to their adoption where 4 is high agreement of importance and 0 means not at all important.

The second figure, Figure 7, shows participants’ levels of agreement on the importance of various technology related factors when considering the adoption of a new technology. Users were asked to rank agreement values where 0 is not at all important and 4 was very important for each category. Users felt cost, ease, safety, and how well the technology meets their needs were most important, while the way it looks and how visible it is to others had more widely spread levels of less importance.

### 4.2. Performance Metrics

Task performance metrics were recorded for completion time and success rates for the drinking and popcorn making tasks. All participants completed eight trials except one, who was only able to complete a partial set due to reaching the maximum allowed testing time across two scheduled visits. This participant contributed one trial for each control mode in the drinking task, one manual control trial in the popcorn task, and two VGS trials in the popcorn task. Therefore, weighted averages and standard deviations were calculated for time across all participants where the weight for every participant reflected the number of that participant’s trials.

Table 3 summarizes completion times and success rates for each subtask in the drinking task. The first two columns show average task times calculated only from successful trials, using a weighted average that excludes failed attempts. The next two columns include all trials, both successful and failed. For tasks such as *Open cabinet* the average time for successful trials is much lower, as it reflects only the subset of participants, five in this study, who were able to complete the task efficiently. In general, VGS subtasks were completed faster than their manual counterparts, with the exception of *Fill drink cup and retract* and *Bring cup to drink*, where task times were comparable. These two subtasks involved a mix of autonomous (robot) and manual (user) actions, as shown in Table 1. For instance, in *Fill drink cup and retract*, the system aligned the cup with the dispenser, but the user controlled the actual filling and retraction process. Similarly, for *Bring cup to drink*, the system moved the cup above the table, but completion of the subtask depended on varied user movement where variable times attribute to a user’s drinking preference over difficulty.

In the drinking task, success rates were over 90% for all subtasks with the exception of the *Open cabinet* task under manual control, which had a 39% success rate. Difficulty of the *Open cabinet* subtask was attributed to the fan-shaped motion trajectory required to open a cabinet door where participants coordinate between X-Y mode and wrist orientation mode. Several participants avoided switching to wrist mode and attempted the motion using only translation, which often resulted in the door bouncing back and incomplete opening.

For the popcorn task, Table 4, four subtasks *Dispense popcorn kernels*, *Open microwave*, *Place jug in microwave*, and *Close microwave*, had similar average completion times between manual and VGS control, differing by less than 15 s. In *Dispense popcorn kernels* and *Open microwave*, both control methods required waiting for precise alignment in VGS in order to initiate a pushing motion. In some VGS trials, participants interrupted the automatic alignment due to impatience, which reduced potential timing advantage. The *Place jug in microwave* and *Close microwave* subtasks were performed manually fully by the user in both conditions, resulting in no true difference of control method.

Most subtasks in the popcorn task had success rates above 90%, with six exceptions. In the manual control condition, *Pour kernels in jug* achieved only 78.2% success, due to a wrist rotation and translation requirement for accurately pouring all kernels. This was further complicated due to lack of depth perception and line of sight obstruction when sitting with limited trunk control. Similarly, *Grasp jug lid* under manual control had a 65% success rate due to the user having difficulty aligning and grasping at an angle.

In the VGS condition, *Grasp jug lid* achieved an improved 87.5% success rate, though three failures occurred. Two were due to the robotic arm approaching the lid from a suboptimal angle based on its previous position, which prevented a clear path to the lid. The third failure was due to an insufficiently secure grasp that resulted in the lid slipping during movement.

Subtasks for *Place lid on jug* were difficult in both control conditions with manual control 56.5%, and VGS at 66.7%. Even in VGS mode, after aligning the lid, the user had to release it manually and ensure proper placement. Inconsistent release strategies, combined with visual alignment difficulties and depth perception challenges that increased given limited trunk control, led to multiple failures in this subtask.

The VGS condition for *Open microwave* had a success rate of 83.3%. Failures in this subtask were often due to wrist misalignment with the fiducial tag. In two cases, participants attempted to rush the process and interrupted the system mid-alignment, causing the push action to be slightly off-center. This led to partial openings that required manual correction. Another failure resulted from a planning error when the arm’s position after the prior subtask created a collision path with the table. The final failure occurred due to lighting conditions; bright sunlight reduced fiducial tag contrast, affecting the accuracy of the arm’s alignment with the microwave button.

### 4.3. Usability Metrics

#### 4.3.1. Mental Workload

**Figure 8:**
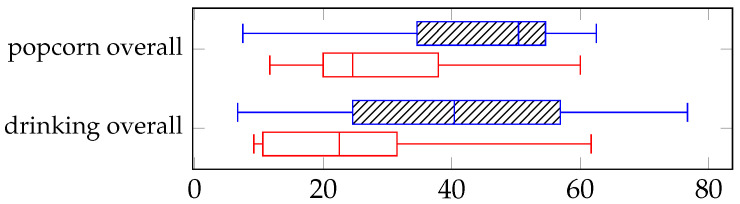
Comparison of overall NASA-TLX scores for the drinking and popcorn task. Manual control represented by blue dashed lines (**top** boxplot) and VGS is the open red box (**bottom** boxplot).

We performed a non-parametric related samples Wilcoxon Signed Rank Test to statistical compare the overall NASA-TLX workload score between control methods, as it served as our primary workload outcome, Figure 8. The overall workload score showed a significant difference (0.019, *p* < 0.05). Median effort ratings were higher in the manual condition (50.4, IQR = 34.6–54.6) compared to the VGS condition (24.6, IQR = 20–37.9) for the popcorn task, and higher in the manual condition (40.4, IQR = 24.6–56.9) compared to the VGS condition (22.5, IQR = 10.6–31.5) for the drinking task.

**Figure 9:**
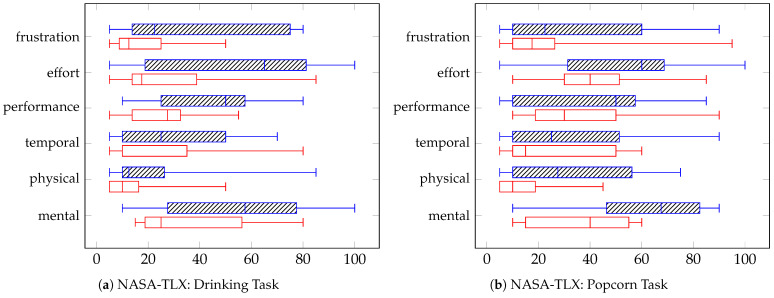
NASA-TLX scores for the drinking and popcorn tasks across each of the individual six dimensions. Manual control is represented by the dashed blue lines (**top** boxplot) and VGS is the red open box (**bottom** boxplot).

We also compared the six NASA-TLX sub-scales between the VGS and Manual control conditions using a Wilcoxon Signed-Rank Test. To account for multiple comparisons, we applied a Bonferroni correction, setting the significance threshold at *p* < 0.0083 (0.05/6). Since none of the sub-scale scores were significant, we reported them visually in Figure 9 to provide further context.

#### 4.3.2. Usability Scale

**Figure 10:**
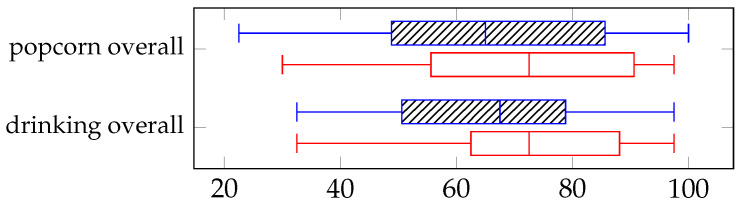
System Usability Score (SUS) for manual control shown as dashed blue lines (**top** boxplot) and VGS control as red open lines (**bottom** boxplot), for each task.

Usability for both controls and both tasks were good and showed no significant differences for the control or task shown in Figure 10. The popcorn task showed median SUS in the manual condition as (65, IQR = 48.8–85.6) compared to the VGS condition (72.5, IQR = 55.6–90.6), and the drinking task showed median SUS in the manual condition as (67.5, IQR = 50.6–78.8) compared to the VGS condition (72.5, IQR = 62.5–88.1).

### 4.4. Qualitative Insights

After study completion, users gave a short exit interview focused on how they envision using the robotic arm in their daily lives and preferences between the manual and vision-guided shared control modes. We used deductive coding based on the structure of the interview questions to group participant feedback. These insights present user perceptions of robotic arm use in everyday life and their control preferences, including perceived strengths and limitations of each method.

#### 4.4.1. ARM Usage

All participants gave examples of using the robotic arm for picking things up and reaching for items (n = 12). Additional task suggestions included holding a cell phone, getting a meal, unloading laundry, gardening, reaching for items in a grocery store, and feeding the dog. Another remarked that having a robotic arm could help in safety situations, “But it is ongoing drama around door openers breaking …I got trapped for seven to eight weeks in my building, unable to get in and out of a building which is a fire safety issue for me and other residents,” (P2).

Most users (n = 9) indicated they would attempt a task with the robotic arm before asking for caregiver assistance, especially when alone or during times when caregiver services were limited or unavailable. One participant remarked how caregiver “service in evening and on weekends is difficult” (P3). Several emphasized the importance of their autonomy, preferring to try the technology before relying on another person.

However, users expressed mixed views about the appropriateness of the robotic arm for more personal care tasks. Although it seems the robotic arm could be useful in safety scenarios (i.e., navigating out of buildings), it also could be a safety concern for tasks which require contact with the individual when they have no feeling. One user explained they would like to try assistive technology for bowel function, but they felt the gripper would be dangerous to use. They said, “bowel care is a huge part of my life …by far the hardest caregiver situation to cover …I have no feeling …if I had technology to assist me I would try it, but I would be scared” (P9). Another user remarked, “I don’t think I would use it for personal care because you might need a person to do that for emotional reasons” (P5), and a third participant remarked, “some things I don’t think any machine could help like getting dressed, or bathing myself, those are my two biggest challenges” (P1).

In general, participants felt the robotic arm was less suitable for tasks requiring fine manipulation, physical contact, or sensitive personal care, but highly valuable for basic reaching and retrieval. Some expressed a conditional preference for caregiver assistance when speed was a priority. As one user summarized, “it’s just the time thing, if my caregiver is right there …but if I was by myself then I would use that,” (P7).

#### 4.4.2. Control Preferences

Preferences were balanced between manual joystick control and VGS: six users preferred VGS control, five preferred manual, and one user said they really liked both. While performance data showed consistent advantages for VGS, control preferences were shaped by more nuanced user priorities such as autonomy, effort, and consistency.

**Figure 11:**
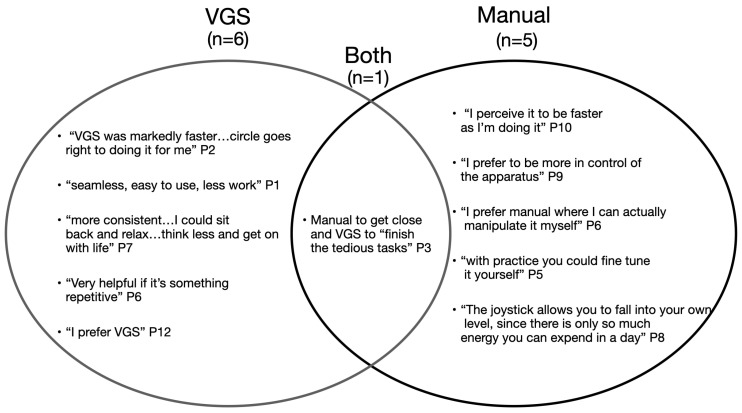
Qualitative quotes illustrating participants’ reasoning for preferring specific control methods.

Strengths and weaknesses of each control mode are shown in Figure 11. Participants who preferred manual control cited a greater sense of control and flexibility. Some found manual control to be more reliable, especially in situations where the autonomous system could not find a path or failed to execute a task. Manual control was also valued for offering more opportunities to practice and refine personal skill. Several users expressed a desire to “beat” the VGS system by completing tasks faster on their own, reflecting a competence and mastery mindset.

In contrast, participants who preferred VGS control emphasized reduced cognitive load and effort. The system was seen as especially helpful for repetitive tasks or when users were fatigued or distracted. One participant mentioned that this is important, especially on days when he is not feeling his best. VGS was also favored for subtasks requiring fine precision, such as pouring and alignment, which some found difficult to perform manually.

Some users saw value in combining both modes, expressing interest in using manual control to get close to a target and VGS to complete more tedious or precise subtasks.

## 5. Discussion

While the VGS system demonstrated objective benefits in performance, and workload reduction, user preferences for control mode were not consistently aligned with these improvements. Performance was improved during VGS control in several subtasks that required coordination of multiple movement dimensions, such as *Opening cabinet* or *Pour kernels into jug*, Section 4.2. These tasks often posed challenging due to depth perception limitations, and the need for precise wrist manipulation. Task success rates, Section 4.2, were also generally higher for VGS control, with notably large performance differences on subtasks such as *Open Cabinet*, *Pour kernels in jug* and *Place lid on jug* where software autonomy could also compensate for perceptual limitations. Quantitative results showed that VGS control significantly reduced overall workload as measured by the NASA-TLX across both tasks Section 4.3.1. Despite these benefits, usability ratings via the SUS, Section 4.3.2, were not significantly different between VGS and manual control, suggesting that participants still found manual control useful. This is also consistent with qualitative findings.

A central insight is that performance metrics alone are insufficient to predict user control preferences. As with other studies, we conclude that user preference for autonomous software with increased assistance is influenced by multiple factors, and performance metrics alone are not good indicators of user preference; this has also been shown in [14,19,20,21,22]. Our findings demonstrate that user preference for robotic arm control modes is multifactorial. Related studies support this. For example, Gervasi et al. [32], evaluated user preferences based on robot speed, perceived control and the number of operational trials. Their study found that operating the robot at a high speed, even though it completes the task faster, made the user feel unsafe. Additionally, when the task is not initiated by the user, the sense of control decreases, reducing user satisfaction. However, users preferred autonomous task initiation once they got familiar with the robot, enhancing efficiency. Styler et al. (2024) [21] also found that for users who preferred manual control, the most cited reason was the sense of control it provided. This highlights perceiving sense of agency is critical in human-robot interaction.

Interviews revealed that six participants preferred VGS, five preferred manual control, and one had no clear preference. Users who favored VGS often cited the reduced need for step-by-step thinking and greater consistency during repetitive tasks. In contrast, manual control was valued for allowing flexibility, personalization, and a greater sense of agency during nuanced actions. Additionally, some participants described trying to “beat” the VGS in speed or preferred the feeling of mastery that came from joystick control. Several users mentioned that VGS felt overly structured, limiting the spontaneity that manual control offered. This again aligns with prior research indicating that perceived control, not just efficiency, plays a major role in assistive technology acceptance.

Future studies should incorporate additional questions to further explore user experience, such as whether participants feel more efficient with autonomous assistance, their perceived level of control among different operation modes, and user trust. Trust can be enhanced by increasing the transparency of the robot’s actions through feedback and sound. Also, objective evaluation metrics, including physiological measures such as heart rate variability and electrodermal activity, could be considered to quantitatively assess user stress levels [33].

### 5.1. Factors That Contributed to Task Difficulty

Depth perception and wrist modes increased task difficulty. For example during task execution, participants struggled with visual alignment when pouring kernels, asking how far they were from the jug, “I can’t see all the angles I need to see” (P2). Similar issues arose when aligning the lid with the jug, where irregular lid geometry and loose grasps led to failed attempts. Participants also experienced confusion differentiating wrist rotation and wrist orientation modes, noting that wrist movements did not match their intuitive expectations from using their own hands. Users said “x-y mode feels more natural like when I move my wheelchair” (P7); “the wrist ones are really hard” (P7).

As shown in the success rate table, several subtasks challenged both control methods. For VGS, failures occurred during tasks with shared user and software control that were also challenging in manual modes. Some failures were due to wrist orientations requiring additional alignment with fiducial tags, or environmental conditions delaying tag detection. Since there is a tradeoff of control between the user and the system both during subtasks and within the transitions between subtasks, there were instances where the arm was in a position that made it difficult to complete the next subtask automatically. Based on these examples, a future system could notify the user with more information such as ’unable to find path’ or give feedback such as ’move end effector to center of table’. This points to an opportunity for future systems to provide more transparent communication and corrective guidance during transitions.

Several factors contributed to task difficulty across both control conditions. These include: (1) the need to manipulate the robotic arm in non-ideal reach zones (e.g., *Open cabinet*), requiring coordinated XY and wrist mode control; (2) the complexity of grasping irregularly shaped objects (e.g., *Grasp jug lid*); (3) depth perception and alignment challenges (e.g., *Pour kernels in jug*, *Place lid on jug*); and (4) individual operational ability, such as understanding control modes and switching between them efficiently. Recognizing these task characteristics is essential for future system design and evaluation frameworks, as they directly influence user performance and preference.

Wrist mode confusion and depth perception challenges emerged as two of the most common barriers to effective arm use. Selvaggio et al. (2021) also revealed that the rotation mode significantly contributes to more decreased performance and increased difficulty than the translational mode (moving on an X, Y, Z plane) [18]. To address these limitations, future systems should consider offering more wrist-specific training and incorporating feedback mechanisms such as distance sensors, augmented visuals, or haptic cues to help users understand when objects are aligned or properly grasped. Several studies have improved the feasibility of these methods. Styler et al., (2025) [34], implemented visual and auditory feedback using four different conditions: continuous light feedback (a blinking white light with variable rate depending on the proximity to the goal); discrete light feedback (white when far from target, yellow when midway, red when close but unaligned, and green meaning action executable); continuous sound feedback (beeping rate increased when robot closer to the goal); discrete sound feedback (tone changes far, mid, and near, and culminating in a dinging sound meaning the action is executable). Results showed that discrete color feedback was the most preferred overall, and continuous beeping was the most preferred sound-based method. Recent developments in augmented reality also offer promising approaches to improve user training and experience. These include providing force feedback to the user [35], and providing a holographic end-effector to preview motion trajectories of the robot [36], allowing users to visualize and verify robot motions before execution.

### 5.2. Context-Dependent Preferences for Arm Usage

Participants also discussed how their preferences might vary by context, Section 4.4.1. Some reported that they would attempt a task with the robotic arm first, particularly when alone or when caregiver availability was limited. However, they also emphasized that in time-sensitive situations, they might defer to a caregiver. The robotic arm was generally not seen as appropriate for tasks involving personal care, emotional support, or close physical contact. Tasks requiring fine motor control, depth alignment, or sensitive tactile feedback (e.g., placing a lid, dressing, or bathing) were seen as less feasible. Conversely, tasks such as reaching, retrieving, and repetitive actions like pushing a button or grabbing an object were viewed as ideal uses. Beaudoin et al. (2019) [37], conducted interviews with long-term users of the JACO robotic arm, who had used the arm for at least six months. Most participants expressed overall satisfaction with the JACO arm and reported positive psychosocial impacts. Users commonly used JACO for drinking, meal preparation, and eating. Future work could explore the integration of autonomous functionalities into the robotic arm to assess whether the burden of complex manipulation could be reduced in real-time, everyday scenarios.

### 5.3. The Potential Value of Systems with Adaptive Control

Participants frequently described how control preference was shaped not just by performance, but also by how easily they could envision the arm fitting into their lives. Users that preferred VGS remarked that it might be more useful for repetitive tasks, tasks where you do not want to think step by step. In other cases, VGS was perceived as too structured, and it was not always clear to users how they could modify or customize its behavior. Another user discussed how once the novelty of manually controlling the arm wears off, they could imagine tasks where they would prefer efficiency, such as hosting a friend (like getting them a drink) and would prefer VGS over attempting the task again. One user did say that “VGS could be a good training tool, especially if there are things you always repeat” (P5). These findings point toward the potential value of adaptive control in training and user-driven customization.

Future research should prioritize longer-term deployments of assistive robotic systems in home or community settings, where users can explore and adapt the technology over time. Studies should also investigate how the setup and configuration process affects user perception of control and autonomy. While brief laboratory studies can demonstrate performance differences, they may not reflect how users balance autonomy, effort, and emotional factors over extended use in real life.

### 5.4. Lessons Learned from the Study Design

A valuable contribution is the set of lessons learned in designing and executing a multi-hour, lab-based evaluation with participants who have mobility and upper limb impairments. The original study protocol involved a single six-hour session in which participants completed four trials of each task and two trials per control condition, with randomized task and control order. However, after five participants (whose data were excluded from analysis), it became clear that the original protocol imposed excessive cognitive and physical demands.

Participants reported significant fatigue, and several experienced difficulty sustaining focus and performance throughout the session, even with breaks, snacks, and other supports. In response, we revised the protocol to better accommodate participants’ energy levels and scheduling constraints. Specifically, we made three key changes: (1) sessions were split into two separate visits of approximately three hours each; (2) the number of subtasks in each task was reduced to shorten task duration; and (3) the VGS condition was always presented first to simplify training and ensure participants were familiar with the autonomous features before attempting manual control.

### 5.5. Limitations

In our updated protocol, presenting the VGS control condition first removed full counterbalancing and introduced potential order effects, such as improved performance in the manual trials due to task familiarity. It may also have framed VGS as the default, potentially influencing user perceptions. However, we believe this sequence is justifiable. Unlike drug trials, assistive technology evaluation depends not only on performance but also on user experience and acceptance in realistic use contexts. Notably, VGS still outperformed manual control despite being presented first, and participant preferences remained mixed, suggesting order effects did not drive the results. Previous studies have shown that order effect results in a learning effect where participant become more efficient with more manual task trials [34]. While the fixed order of conditions limits experimental control, it prioritized participant well-being and operational feasibility. We report findings from the revised protocol to reflect these practical considerations.

This study offers only a brief window into how novice users interpret and interact with assistive robotic arms. As such, their ability to fully evaluate the tradeoffs between manual and autonomous modes may have been limited. As novices, this was users first experience using a robotic arm. Initial enthusiasm or unfamiliarity may have skewed both their performance and subjective feedback, especially in the manual condition where mode-switching required learning and adaptation. Some participants noted that manual control became more comfortable with practice, suggesting that longer-term familiarity could change their preferences. Conversely, users may not have fully grasped the customization or long-term potential of the VGS system within the limited study duration, which could under-represent its usability in daily life.

While the sample included a diverse range of diagnoses and upper limb abilities, it was relatively small (n = 12), limiting the ability to detect statistically significant differences or generalize findings to broader populations. Moreover, all participants completed the study in a controlled lab environment using predefined tasks. This setting introduced constraints that may not reflect real-world use. For example, because the robotic arm was mounted to a table rather than to the participant’s wheelchair, users were unable to reposition themselves, making it more difficult to see the end effector from their seated perspective. The lab environment also limits real-world variability. As such, user behavior, preferences, and performance may differ in everyday settings where autonomy, caregiver availability, environmental conditions, and task urgency vary considerably. Although our sample size limits generalizability, the combination of performance trends and interview feedback offers valuable insights.

## 6. Conclusions

This study examined how power wheelchair users interact with assistive robotic manipulators during multi-step tasks using a VGS control system. While VGS reduced cognitive workload and improved performance for many, user preferences were mixed, revealing that technical performance does not always align with user satisfaction. Qualitative feedback showed that factors such as task type, user context, and individual confidence play critical roles in shaping the perceived value of robotic assistance. Usability challenges, such as depth perception, wrist mode confusion, and alignment difficulties highlight opportunities for design improvement. Future research should explore longer-term, in-home studies to understand how user preferences evolve over time, particularly in relation to system configuration, user autonomy, and integration into daily life.

## Figures and Tables

**Table 1:** VGS action descriptions.

Drinking Task	
SubTask	Control Handoffs
Grasp cabinet handle	1 (User): Move joystick to cabinet tag; when circle appears, press to hand off to robot.
	2 (Robot): Grasp cabinet handle.
Open cabinet	1 (Robot): Open cabinet and release handle.
Grasp drink cup	1 (User): Move joystick to cup tag; when circle appears, press to hand off to robot.
	2 (Robot): Grasp cup and retract.
Fill drink cup and retract	1 (User): Move joystick to jug tag; when circle appears, press to hand off to robot.
	2 (Robot): Align cup with jug.
	3 (User): Push cup against jug tap to dispense water, retract cup, and press screen to continue.
Bring cup to drink	1 (Robot): Move cup above center of table.
	2 (User): Bring cup to mouth, drink, retract, and press screen to continue.
Place cup on table	1 (Robot): Move cup over table.
	2 (User): Release cup.
**Popcorn Task**	
**SubTask**	**Control Handoffs**
Dispense popcorn kernels	1 (User): Move joystick to dispenser tag; when circle appears, press to hand off to robot.
	2 (Robot): Dispense kernels; align with cup.
Grasp popcorn cup	1 (Robot): Grasp popcorn cup and retract.
Pour kernels in jug	1 (Robot): Move cup over jug, pour kernels, and retract.
Place cup on table	1 (Robot): Move cup over table.
	2 (User): Release popcorn cup.
Grasp jug lid	1 (User): Move joystick to lid tag; when circle appears, press to hand off to robot.
	2 (Robot): Grasp lid and place over jug.
Place lid on jug	1 (User): Further align lid and release.
Open microwave	1 (User): Move joystick to microwave tag; when circle appears, press to hand off to robot.
	2 (Robot): Push microwave button and retract.
Grasp jug	1 (User): Move joystick to jug tag; when circle appears, press to hand off to robot.
	2 (Robot): Grasp jug and lift from table.
Place jug in microwave	1 (User): Move jug into microwave and release.
Close microwave	1 (User): Move arm to close microwave.

**Table 2:** Demographic information and SCI-FI score.

Participant	Diagnosis	Age	Gender	SCI-FI T-Score (Mean 50, Std 10)
P1	Muscular Dystrophy	38	Male	58.5
P2	Cerebral Palsy	53	Male	40.0
P3	Muscular Dystrophy	76	Male	59.5
P4	Cerebral Palsy	73	Male	27.6
P5	Post-Polio Syndrome	77	Female	41.0
P6	Spinal Cord Injury (SCI)	54	Female	51.3
P7	SCI	30	Female	49.2
P8	Inflammatory Demyelinating Polyneuropathy	65	Male	46.1
P9	SCI	61	Male	54.3
P10	SCI	20	Male	57.5
P11	Spinal Stenosis	35	Male	52.3
P12	Cerebral Palsy	47	Male	62.6

**Table 3:** Task Completion Time and Success Rates of the Drinking Task.

Drinking Task						
	Task Time (s) Only Success Manual	Task Time (s) Only Success VGS	Task Time (s) with Failure Manual	Task Time (s) with Failure VGS	Success Rate Manual	Success Rate VGS
Grasp cabinet handle	50.7 ± 27.4	42.3 ± 21.5	72.7 ± 50.3	42.3 ± 21.5	91.3%	100%
Open cabinet	31.3 ± 16.5	15.5 ± 4.7	194.8 ± 130.7	15.5 ± 4.7	39.1%	100%
Grasp drink cup	106.9 ± 61.6	47.9 ± 12.6	115.3 ± 65.5	59.7 ± 37.0	95.7%	95.7%
Fill drink cup and retract	65.4 ± 28.2	78.7 ± 27.7	65.4 ± 28.2	88.3 ± 39.9	100%	95.7%
Bring cup to drink	65.6 ± 28.3	52.9 ± 29.1	75.8 ± 37.1	74.5 ± 53.6	95.7%	91.3%
Place drink cup on table	61.7 ± 36.5	36.9 ± 16.8	72.1 ± 59.7	59.8 ± 56.1	95.7%	91.3%

**Table 4:** Task Completion Time and Success Rates of the Popcorn Task.

Popcorn Task						
	Task Time (s) Only Success Manual	Task Time (s) Only Success VGS	Task Time (s) with Failure Manual	Task Time (s) with Failure VGS	Success Rate Manual	Success Rate VGS
Dispense popcorn kernels	63.2 ± 27.9	53.7 ± 21.5	63.2 ± 27.9	53.7 ± 21.5	100%	100%
Grasp popcorn cup	82.5 ± 37.4	17.8 ± 3.4	91.9 ± 50.2	17.8 ± 3.4	95.7%	100%
Pour kernels in jug	134.8 ± 58.4	29.5 ± 2.6	169.8 ± 82.5	29.5 ± 2.6	78.2%	100%
Place popcorn cup on table	74.6 ± 38.1	29.2 ± 29.6	84.4 ± 47.6	29.2 ± 29.6	95.7%	100%
Grasp jug lid	114.0 ± 59.1	84.1 ± 23.6	171.3 ± 86.1	111.1 ± 61.8	65.2%	87.5%
Place lid on jug	82.7 ± 36.4	67.7 ± 38.5	180.3 ± 80.8	118.9 ± 68.2	56.5%	66.7%
Open microwave	118.9 ± 68.2	99.3 ± 40.9	118.9 ± 68.2	132.7 ± 53.8	100%	83.3%
Grasp jug	104.9 ± 56.4	75.9 ± 24.4	104.9 ± 56.4	75.9 ± 24.4	100%	100%
Place jug in microwave	77.8 ± 38.5	98.1 ± 42.8	87.5 ± 47.0	98.0 ± 42.8	95.7%	100%
Close microwave	57.3 ± 38.5	55.4 ± 29.4	57.3 ± 38.5	55.4 ± 29.4	100%	100%

## Data Availability

The data presented in this study are available on request from the corresponding author. The data are not publicly available due to being restored by the US Department of Veterans Affairs, and are subject to the approval of the relevant authority.
